# Exploring Social Bonds and Well-Being in Young Adults during and beyond the COVID-19 Pandemic

**DOI:** 10.3390/jcm12237298

**Published:** 2023-11-24

**Authors:** Emanuela Mari, Alessandro Quaglieri, Micaela Di Consiglio, Alessandro Couyoumdjian, Clarissa Cricenti, Giulia Lausi, Alessandra Pizzo, Vincenzo D’Amato, Sabina D’Amato, Emilia Anna Vozzella, Antonietta Ferrara, Anna Maria Giannini, Jessica Burrai

**Affiliations:** 1 Department of Psychology, Sapienza University of Rome, Via dei Marsi 78, 00185 Rome, Italy; e.mari@uniroma1.it (E.M.); alessandro.quaglieri@uniroma1.it (A.Q.); micaela.diconsiglio@uniroma1.it (M.D.C.); alessandro.couyoumdjian@uniroma1.it (A.C.); clarissa.cricenti@uniroma1.it (C.C.); giulia.lausi@uniroma1.it (G.L.); alessandra.pizzo@uniroma1.it (A.P.); annamaria.giannini@uniroma1.it (A.M.G.); 2 Direzione Generale, A. O. U. San Giovanni e Ruggi d’Aragona, Largo Città d’Ippocrate, 84131 Salerno, Italy; direzione.generale@sangiovannieruggi.it (V.D.); antonella.ferrara@sangiovannieruggi.it (A.F.); 3 Direzione Sanitaria, Servizio di Psicologia Clinica Ospedaliera, A. O. U. San Giovanni e Ruggi d’Aragona, Largo Città d’Ippocrate, 84131 Salerno, Italy; sabina.damato@sangiovannieruggi.it (S.D.); direzione.sanitaria@sangiovannieruggi.it (E.A.V.)

**Keywords:** social connectedness, older adolescent, depression, anxiety, stress, health emergency, life quality

## Abstract

Background: Young adults, aged between 17 and 25 years, experienced a strong impact on both their mental health and well-being due to COVID-19. Indeed, they were simultaneously faced with the normative tasks of their age and stressors associated with the long-lasting COVID-19 pandemic. This study offers further insights into the perceptions of the well-being (stress, anxiety, and depression) and social bonds in young Italian adults during and after the COVID-19 pandemic. Methods: The first survey was conducted between December and February 2021 (i.e., during the second wave in Italy) and included a total sample of 347 participants. The second survey took place between April and May 2022 (i.e., at the end of the health emergency in Italy) and consisted of a total sample of 313 participants. Results: T-tests, correlations, and linear regressions were performed. Overall, our findings showed an increased mood disturbance was positively correlated with having contracted COVID-19 and negatively associated with social connectedness. Furthermore, social assurance was found to negatively predict mood disorders during COVID-19. Conclusion: While numerous studies have focused on mental health, there has been limited exploration of protective factors, which could represent a different perspective that emphasizes individuals’ resources rather than their vulnerabilities.

## 1. Introduction

### 1.1. During and beyond the COVID-19 Pandemic

At the end of February 2020, Italy faced an outbreak of coronavirus disease 19 (COVID-19), which spread quickly across most parts of Europe (first wave). After a decrease in the number of cases detected in the summer, Europe faced an emerging second wave of COVID-19 (September 2020–January 2021). In this context, the Italian government established a partial lockdown based on a new tiered system, classifying some areas with the highest rates of COVID-19 as high-risk red zones and maintaining some preventive measures (e.g., wearing a mask and social distancing). Several waves of COVID-19 followed, causing several hundred thousand infected people and thousands of deaths. Until 31 March 2022, when the Italian Government established the end of the COVID-19 state of emergency. The path for the gradual return to ordinary life included a number of stages:

1. The end of the colored zone system (i.e., red, orange, yellow, white) that indicated the risk classification of regions and the restrictive measures to be implemented according to the color.
2. The gradual phasing out of the basic and enhanced green pass.
3. The elimination of precautionary quarantines.

### 1.2. COVID-19’s Impact on Young Adults

Impacts on people’s lives caused by COVID-19 have been numerous for different age groups (e.g., [1,2,3]), populations (e.g., [4,5]), and genders (e.g., [6,7]). 

Young adults between the ages of 17 and 25 experienced greater impairments with respect to both mental health and well-being. In fact, young adults were simultaneously faced with the normative tasks of their age (e.g., building a professional career, seeking financial independence, building important social and romantic relationships) and the stressors associated with the long-lasting COVID-19 pandemic. As emphasized by the Transitions Overload Model [8], young people are unable to handle multiple stressors simultaneously, and the accumulation of multiple stressors reduces overall well-being. Stress overload can hinder individuals’ ability to cope and increase mental health problems (e.g., [9]). A systematic literature review developed by Kauhanen and colleagues [10], conducted on 21 studies from 11 countries, showed the longitudinal worsening of symptoms for several mental health outcomes, especially for adolescents and young people. Hence, different studies conducted during COVID-19 have shown age-related differences: young adults reported higher rates of anxiety, depression, and loneliness during the pandemic (e.g., [11,12]). In addition, regarding gender, a systematic literature review of 23 studies found that women and adolescents showed higher levels of anxiety and depression due to COVID-19 [13]. 

Some studies have also investigated the psychosocial environment of adolescents related to pandemic situations. Increased time spent at home with family seems to have influenced conflict with parents, harsh discipline, and parental control [14,15]. Overall, for example, in Mari and colleagues’ study [16], it was found that during COVID-19 isolation, interpersonal relationships with cohabiting people had an important impact on psychological well-being and, in particular, cohabitation was a mediator on stress, future perspectives, and coping strategies during the COVID-19 lockdown in Italy.

Thus, the pandemic has led to a necessary reduction in social contacts, but, as reported by scientific studies, the psychological sense of community, understood as feeling part of a group, is positively correlated with higher levels of well-being, and is associated with prosocial behavior, civic participation, and the promotion of social capital (e.g., [17,18]).

### 1.3. Social Connectedness and Well-Being

During the COVID-19 pandemic, the social connections of adolescents (defined as a sense of an internal closeness within the social world, including relationships with family, friends, community, school, and neighbors [19]) have played a crucial role in their mental well-being [20]. Social contact plays a critical function in promoting well-being and is particularly vital during periods of stress [21]. Therefore, it is likely that restrictions imposed by lockdowns [22,23,24] and social distancing practices have contributed to increased emotional distress and feelings of loneliness [25,26]. However, these changes also expose adolescents to greater social stressors and heightened sensitivity to social dynamics, both of which are associated with an increased vulnerability to depression [27,28].

Social support is the feeling of being a valued member of a social network in which one feels cared for and is expected to receive help when needed [29]. Social support becomes particularly important when people collectively experience psychological effects of a trauma that affects the entire population (e.g., a mass disaster or pandemic [30]).

Several studies have observed that social support is essential for promoting socio-emotional well-being [31,32], especially in young adults who are transitioning towards greater independence from their parents and increased reliance on their friends for social support and interaction. Friends play a significant role as sources of validation, support, and companionship [33] and can be especially crucial during the shared challenges of a pandemic [25]. Although young adults are certainly more vulnerable to restrictions, they also possess specific resources to cope with physical distance, such as instant messaging, social networks, and video calls [34].

### 1.4. Study Aim and Hypothesis

This study aims to further investigate the perception of well-being (i.e., stress, anxiety, and depression) in young Italian adults (in this paper, we will refer to our sample using this wording) during and after the COVID-19 pandemic. Specifically, we wanted to explore how gender, age, cohabitation, having contracted COVID-19, performing social or sports activity, and social connectedness could affect young people’s mood.

For this purpose, our study aims at highlighting differences in the variables investigated in two different phases: during the first phase (i.e., COVID-19 second wave) and during the second phase (i.e., after the end of the Italian pandemic-related emergency).

Specifically, we expect the following:

**Hypothesis** **1** **(H1).** *Significant differences between the first and second phases in terms of depression, anxiety, stress, social connectedness, and social assurance.*

**Hypothesis** **2** **(H2).** *Significant correlations between DASS-21 and the investigated variables according to the phase of COVID-19.*

**Hypothesis** **3** **(H3).** *The investigated variables will be predictors of mood disorders (in this study, understood as the sum of DASS-21 subdimensions) in the first phase compared to the second-phase groups.*

## 2. Materials and Methods

### 2.1. Materials

The demographic characteristics of gender, age, level of education, social and personal activity (e.g., sport, scout), relationships (e.g., cohabitation), and COVID-19 exposure were collected. Subjects were asked to complete the following self-report measures: the depression, anxiety, and stress scale and the social connectedness scale.

Depression Anxiety Stress Scales Short Version (DASS-21 [35]) is a well-known questionnaire used to assess the constructs of depression, anxiety, and stress. In this study, the Italian version edited by Bottesi of the Depression Anxiety Stress Scales Short Version (DASS-21 [36]) was used. Participants were asked to indicate how much the statement applied to them in reference to the previous week on a 4-point Likert scale (from 0 = Not applicable to me at all, to 3 = Applicable most of the time or very much so). It allows for the detection of three dimensions: depression (e.g., “I felt I was pretty worthless”) including dysphoria, hopelessness, devaluation of life, self-depression, lack of interest/involvement, anhedonia, and inertia; anxiety (e.g., “I felt I was close to panic”) refers to autonomic nervous system arousal, skeletal musculature effects, situational anxiety, and subjective experience of anxious affects; stress (e.g., “I felt that I was using a lot of nervous energy”) relates to the presence of nonspecific arousal levels, difficulty relaxing, nervous excitement, irritability, agitation, hyperactivity, and impatience. The psychometric properties in this study showed a good reliability with Cronbach’s alpha of 0.90 for the total score, a Cronbach’s alpha of 0.88, 0.80, and 0.88 for depression, anxiety, and stress dimension, respectively.

The social connectedness Scale (SCS-16—[37]) is a self-report questionnaire designed to assess an individual’s sense of connection with others in their social context. Developed by Lee and Robbins in 1995, it has found extensive application in both research and clinical contexts. Comprising 16 items, the SCS evaluates distinct features of social connectedness, which encompass: Belongingness, Closeness, Support, Satisfaction. To complete the SCS, respondents rate each item on a 6-point Likert scale, ranging from 1 (strongly disagree) to 6 (strongly agree). The factorial structure showed two dimensions: social connectedness (e.g., I feel disconnected from the world around me); and social assurance (e.g., I feel more comfortable when someone is constantly with me). The total score is obtained by summing the scores for all 16 items. The higher the score on the SCS dimensions is, the higher the perception of social connectedness is. Additionally, the SCS has been used in clinical settings to evaluate social connectedness in individuals dealing with mental health conditions such as depression and anxiety. In this study, we used an Italian validation of the SCS scale that showed good reliability, with a Cronbach’s alpha of 0.92 for social connectedness and with an alpha of 0.79 for social assurance.

### 2.2. Participants

The study used an observational cross-sectional design. The first convenience sample was invited to participate in our anonymous online survey between December and February 2021, and consisted of a total sample of 347 participants. A total of 36.0% were male (N = 125), and the ages ranged from 18 to 24 (M = 20.53, SD = 1.72). With respect to the educational level, (N = 314) were high school, (N = 8) were middle school and, (N = 25) were university students. Concerning having contracted COVID-19 the 54.2% (N = 188) stated “Yes”, also, the 34.3% (N = 119) reported cohabiting with someone (i.e., regardless of sentimental relationship), and lastly, the 88.2% (N = 306) reported not doing any social and personal activity (e.g., sport).

The second sample was invited to participate between April and May 2022 (at the end of health emergency in Italy) and consisted in a total sample of 313 participants. A total of 33.5% were male (N = 105), and the ages ranged from 17 to 25 (M = 20.64, SD = 1.91). With respect to the educational level, (N = 266) were high school, (N = 36) were middle school, and (N = 9) were university students. Concerning having contracted COVID-19, 38% (N = 119) stated “Yes”, also, the total sample (N = 313) reported cohabiting with someone (i.e., regardless of sentimental relationship), and lastly, the 57.8% (N = 181) reported doing social and personal activity.

### 2.3. Data Analysis

Statistical analyses were conducted using the Statistical Package for Social Science (SPSS; version 26.0; IBM SPSS, Armonk, NY, USA). Firstly, distributions of all data were verified for normality, and the internal consistency of the instruments was tested using Cronbach’s alpha. Descriptive statistics with means and standard deviations were performed and chi-square was used to test differences for gender (i.e., nominal variable). We conducted a multivariate analysis of variance (MANCOVA) with well-being measures (i.e., DASS-Stress, DASS-Anxiety, DASS-Depression, social connectedness, social assurance), as dependent variables and phase (i.e., during second wave of the COVID-19, post-COVID-19 pandemic) as a fixed factor and having contracted COVID-19 as a covariate. Bonferroni correction adjustment was used for multiple comparisons. Having contracted COVID-19 was included as a dichotomous covariate (covariate was evaluated in the model with a value of 0.47) to control for possible differences in the sample. The parametric assumptions of the equality of variance of scores between the groups were checked by means of variance ratio tests and Levene’s tests. Pearson’s correlations and regression analyses were performed to explore the relationships between the dependent variable (i.e., DASS-21 total score) and the verified predictors. Statistical significance was defined as *p* < 0.05. All statistical analyses were performed on de-identified data.

### 2.4. Procedure

Data were gathered through the online survey platform Qualtrics in two different phases; in the first phase (i.e., during second wave of the COVID-19 pandemic) data collection started on 18 December 2020, and concluded on 5 February 2021. The second phase (i.e., post-COVID-19 pandemic, at the end of health emergency in Italy) started on 1 April 2022, and concluded on 26 May 2022. Regardless of the phase, the same survey was distributed via social media channels and the official website of the university. Participants provided their consent by digitally signing an informed consent form and were reassured that their responses would remain anonymous and would only be used for the purposes of the current research. Afterwards, participants were invited to complete questionnaires. The study encompassed an examination of demographic characteristics (i.e., gender, age, level of education), exposure to COVID-19, mood disorder and how they feel connected to others in their social environment. This study was conducted in accordance with the ethical principles outlined in the Declaration of Helsinki and received approval from the Institutional Review Board of the Department of Psychology at “Sapienza” University of Rome (protocol number 0002195/18-12-2020).

## 3. Results

There were no statistically significant results regarding the gender (*p* = 0.514) and age (*p* = 0.455) between the groups. All the differences between the groups in the clinical variables are presented in Table 1. There was a difference in the DASS-21 stress index (F_(1,660)_ = 7.63, *p* < 0.01, ή^2^ = 0.01), in which participants during the COVID-19 phase showed a higher mean (M = 9.66, SD = 4.39) than the post-COVID phase (M = 8.65, SD = 4.47); (M = 0.966, SE = 0.350, *p* < 0.01), there was difference in DASS-21 anxiety index (F_(1,659)_ = 50.17, *p* < 0.001, ή^2^ = 0.07) in which post-COVID-19 phase participants reported a higher mean (M = 6.17, SD = 4.0) than during the COVID-19 phase (M = 4.10, SD = 3.49); (M = 2.09, SE = 0.296, *p* < 0.001), there was also a difference in social connectedness (F_(1,660)_ = 12.67, *p* < 0.001, ή^2^ = 0.01), in which post-COVID-19 participants showed a higher mean (M = 36.22, SD = 9.04) than during COVID-19 (M = 33.85, SD = 9.67); (M = 2.63, SE = 0.739, *p* < 0.001). There were no statistically significant results for the DASS-21 Depression index (*p* = 0.079) and for social assurance (*p* = 0.166). There was, instead, a between-subjects effect of the having contracted COVID-19 covariate on social connectedness (F_(1,660)_ = 4.51, *p* < 0.05, ή^2^ = 0.003).

In order to investigate whether there were significant relationships between the descriptive characteristics, the DASS-21 Scale and social connectedness scale and bivariate correlations were conducted using Pearson’s r coefficient (for variables that were normally distributed) for each group (i.e., during and post-COVID-19 phase) separately. All correlations are reported in Table 2.

With respect to the during COVID-19 phase group, age was negatively correlated with DASS-21 depression (r = −0.129; *p* < 0.05), and gender was negatively correlated with DASS-21 Stress (r = −0.199; *p* < 0.01), DASS-21 Anxiety (r = −0.155; *p* < 0.01), and DASS-21 TOT (r = −0.160; < 0.01). Having contracted COVID-19 was positively correlated with DASS-21 Stress (r = 0.105; *p* < 0.05). Social connectedness was negatively correlated with DASS-21 Stress (r = −0.336; *p* < 0.01), DASS-21 Anxiety (r = −0.290; *p* < 0.01), DASS-21 Depression (r = −0.502; *p* < 0.01), and DASS-21 TOT (r = −0.445; < 0.01). Social assurance was negatively correlated with DASS-21 Stress (r = −0.208; *p* < 0.01), DASS-21 Anxiety (r = −0.183; *p* < 0.01), DASS-21 Depression (r = −0.258; *p* < 0.01), and DASS-21 TOT (r = −0.254; < 0.01).

Concerning the post-COVID-19 phase, gender was negatively correlated with DASS-21 Stress (r = −0.240; *p* < 0.01), DASS-21 Anxiety (r = −0.249; *p* < 0.01), DASS-21 Depression (r = −0.204; *p* < 0.01), and DASS-21 TOT (r = −0.244; < 0.01). Social connectedness was negatively correlated with DASS-21 Stress (r = −0.407; *p* < 0.01), DASS-21 Anxiety (r = −0.456; *p* < 0.01), DASS-21 Depression (r = −0.510; *p* < 0.01), and DASS-21 TOT (r = −0.484; < 0.01). Social assurance was negatively correlated with DASS-21 Stress (r = −0.133; *p* < 0.01), DASS-21 Anxiety (r = −0.159; *p* < 0.01), DASS-21 Depression (r = −0.136; *p* < 0.01), and DASS-21 TOT (r = −0.150; < 0.01). All participants reported having a relationship, therefore, the variable cannot be computed in the correlation analysis (i.e., the variable is constant).

A linear regression was performed for each group to identify the predictors of the DASS-21 total score. All variables were removed from the set of predictors that showed excessive correlations (>0.70) between them and compromised the assumption of no multicollinearity.

Overall, in the during COVID-19 group (Table 3), the regression analysis yielded a final model of five predictors, which explained 28.4% of the variability (F_(5,346)_ = 27.02 *p* < 0.001). The Maximum Variance Inflation Factor (VIF) for this model was 1.2, suggesting no significant multicollinearity (i.e., the predictors in the regression were not highly intercorrelated), and indicating that the estimates of the regression coefficients of the model were stable. The model identifies gender as a significant covariate (β = −2.92, SE = 0.130, *p* < 0.01), indicated by the DASS-21 female participants’ score. Also, both the social connectedness and Assurance had a strong association with DASS-21 (β = −0.482, SE = 0.052, *p* < 0.001; β = −0.357, SE = 0.070, *p* < 0.001, respectively), and those who reported lower scores had a more unfavorable DASS-21 outcome. Lastly, reporting having contracted COVID-19 (β = 2.16, SE = 1.09, *p* < 0.05) was significantly associated with the DASS-21 total score.

With respect to the post-COVID-19 phase group (Table 4), the regression analysis yielded a final model of five predictors which explained 27.7% of the variability (F_(5,312)_ = 23.50 *p* < 0.001). The maximum VIF for this model was 1.0, suggesting no significant multicollinearity. Also, in the post-COVID-19 group, gender showed an effect as a covariate (β = −5.03, SE = −0.193, *p* < 0.01), with the male gender seeming to have more favorable scores in DASS-21. In this model, only social connectedness was strongly associated with DASS-21 (β = −0.613, SE = 0.068, *p* < 0.001).

In summary, gender, social connectedness and Assurance and reporting having contracted COVID-19 were significant predictors during COVID-19 phase, while only both gender and social assurance seemed to expose individuals to developing mood disorders in post-COVID-19 phase.

## 4. Discussion

The present study examined the differences, during and after the COVID-19 pandemic, in the perceptions of well-being (i.e., stress, anxiety, and depression) and social support (i.e., social connectedness and social assurance) in young Italian adults. In particular, the research underlined the specific characteristics of the two groups in the development of mood disorder, investigating gender, age, cohabitation, COVID-19, social and personal activity, and social connectedness.

In general, increased mood disorder has been shown to be positively related to having contracted COVID-19, and negatively associated with social connectedness.

Our first hypothesis (H1) was partially confirmed. In the recent meta-analysis by Delpino and colleagues [38] conducted on 194 studies, the results suggested that one in three people worldwide experienced anxiety disorders during the COVID-19 pandemic. Uncertainty, disruptions to daily routines, and concerns about health and well-being during the COVID-19 pandemic likely explain the increase in generalized anxiety. Indeed, many studies have historically shown that uncertainty, as a common feature of the threat context, can elicit fear and anxiety (e.g., [39]). In this regard, the fear of the unknown has been defined as an “individual’s propensity to experience fear caused by the perceived absence of information at any level of consciousness or processing point” [40]. Therefore, fear is present-oriented and relatively certain, whereas anxiety is future-oriented and relatively uncertain. This is in line with our data, which suggested a greater level of anxiety at the end of the pandemic. Towards social connectedness, our data show a statistically significant difference between the second wave and the period after the pandemic, with a higher level of sociality in the second phase of administration, when the restrictions had finished. Previous studies agree with our findings, for example, in the study by Nitschke and colleagues [41], greater social connectedness during the period of social isolation was associated with lower levels of perceived stress, and general and COVID-19-specific concerns. Finally, DASS_21 depression and social assurance were not statistically significant, since the sample of young adults (i.e., ages 17–25) may have had the opportunity to have social relationships with others (e.g., through electronic devices) facilitating the perception of feeling a part of a group that serves as a tool to mitigate the possible development of depressive symptoms.

Our second hypothesis (H2) was partially confirmed. In fact, the results underlined that mood disorders were negatively associated with social connectedness and social assurance in both phases, while female gender was positively correlated with all dimensions of DASS-21, except DASS-21 depression, in the “during COVID-19” phase. Having contracted COVID-19 was positively correlated with DASS-21 Stress only in the first phase of our research, and age was negatively related to DASS-21 Depression in the “during COVID-19” phase. These results may be in line with the current literature [25,26], but there are not many studies that have investigated the relationship between social connectedness and mood disorder, especially in young adults. Although depression levels seem to decrease with increasing age in the first phase of our study, it would appear that young adults have adapted to limitations in people-to-people relationships [42]. This social adaptability probably reflects their generational advantage in electronic communication. In fact, electronic communication (both in terms of frequency and satisfaction) with friends during the second year of the pandemic was associated with less loneliness, while more satisfying electronic contact was also related with less social and generalized anxiety and depressive symptoms [25]. It is interesting to note from our results that females seem to be more affected by mood-related distress in both phases of the research. This finding is in line with previous research, in which the prevalence of depression, anxiety, and stress in females was higher than in males [6,13,43]. Finally, having contracted COVID-19 appears to be a positively stress-related factor only in the early phase, as also revealed in the study by Burrai and colleagues [42]. This is also consistent with the effects on mood experienced during the pandemic phase.

Our third hypothesis (H3) was partially confirmed. The regression analysis showed that both “during” and “post” COVID-19 there is a common pattern in the prediction of mood disorders found in gender and social connectedness. Specifically, the female gender [6,13,43], in both phases, reported higher levels of mood-related distress, as well as low levels of social connectedness [25]. The variables that distinguish the two phases are social assurance and having contracted COVID-19, which are significant only in the survey conducted “during” COVID-19. Social assurance is described as a general need for reassurance from at least one or more people for a sense of belonging; this aspect can be assumed to be more prominent during the pandemic-related periods of isolation, as opposed to when one has returned to routine social relationships. By itself, having contracted COVID-19 is related with possible psychological distress, as reported by Mosiolek and colleagues [44], who reported the co-occurrence of physical and psychiatric symptoms in people with COVID-19 infection. The fact that there is a difference between the two phases can be assumed to be related to the uncertainty of outcomes and consequences for one’s own and others’ health, which is typical of the early stages of the pandemic. In the study conducted by Chudzicka-Czupała and colleagues [45], it emerged that factors such as concern about the lack of healthcare, personal health status, the likelihood of survival in case of COVID-19 infection, and the health status of family members in case of COVID-19 infection, were significantly associated with higher scores in the DASS-21. Finally, with regard to age, our results partially confirmed the data that had already emerged in the literature: only the correlation analysis in the first phase (i.e., during COVID-19) showed a negative correlation between age and DASS-21, as already emerged in previous studies [35,46], while, in the regression models, age was not among the predictors in either stage.

However, the study has some limitations. First, it is limited by the use of online surveys and the use of self-reported questionnaires, which may have influenced the findings through well-known biases, including method biases and social desirability biases. Additionally, the study was limited by the relatively small sample size and was conducted using the online convenience sampling strategy without random sample selection. Finally, another potential limitation of this study, which limits its generalizability, is not having assessed pre-existing mental health conditions as a control variable; in fact, as evidenced by the literature [47], pre-existing conditions may be associated with an increased risk of mental health complications such as increased anxiety and depression and lower well-being during the COVID-19 pandemic. Future studies should ensure the evaluation of possible relationships between pre-existing conditions and mental health complications during a possible future new pandemic to assess the most vulnerable populations more accurately.

## 5. Conclusions

The pandemic has led to a substantial increase in the involvement of the scientific community. While numerous studies have focused on mental health, there has been limited exploration of protective factors, which could represent a different perspective that emphasizes individuals’ resources rather than their vulnerabilities. Additionally, few studies have examined variables related to whether or not individuals have contracted COVID-19. Therefore, our aim was to investigate both the aspects of mood disorders and the role of social support in emerging adulthood, aligning with the previous literature [25,31,32]. Overall, these results offer new insights into the role of social support in preserving well-being when faced with a public health threat that necessitates significant changes in daily interactions during a vulnerable phase of life, such as early adulthood.

## Figures and Tables

**Table 1:** Differences between groups on age, DASS-21, and social connectedness scale.

	Groups	M	SD	F	*p*	ή^2^
DASS-21 Stress	During COVID-19	9.66	4.39	7.63	0.006	0.011
	Post COVID-19	8.65	4.46			
DASS-21 Anxiety	During COVID-19	4.10	3.49	50.17	0.001	0.071
	Post COVID-19	6.17	3.99			
DASS-21 Depression	During COVID-19	7.51	4.62	3.10	0.079	0.005
	Post COVID-19	6.89	4.53			
Social Connectedness	During COVID-19	33.85	9.67	12.67	0.001	0.019
	Post COVID-19	36.22	9.04			
Social Assurance	During COVID-19	27.22	7.15	1.92	0.166	0.003
	Post-COVID-19	27.91	7.70			

**Table 2:** Correlation analyses for each group.

Phase		DASS-21 Stress	DASS-21 Anxiety	DASS-21 Depression	DASS-21 TOT
During COVID-19 (N = 347)	Age	−0.039	−0.083	−0.129 *	−0.098
	Gender	−0.199 **	−0.155 **	−0.068	−0.160 **
	Education	0.005	−0.014	−0.022	−0.022
	COVID-19	0.105 *	0.060	0.000	0.062
	Relationship	−0.062	0.001	−0.065	−0.052
	Activity	−0.004	0.031	−0.098	−0.034
	Social connectedness	−0.336 **	−0.290 **	−0.502 **	−0.445 **
	Social assurance	−0.208 **	−183 **	−0.258 **	−0.254 **
Post COVID-19 (N = 313)	Age	0.026	0.038	0.026	0.031
	Gender	−0.240 **	−0.249 **	−0.204 **	−0.244 **
	Education	0.047	0.023	−0.010	0.028
	COVID-19	−0.057	−0.027	−0.029	−0.040
	Relationship	-	-	-	-
	Activity	−0.078	−0.074	−0.075	−0.080
	Social connectedness	−0.407 **	−0.456 **	−0.510 **	−0.484 *
	Social assurance	−0.133 *	−0.159 **	−0.136 *	−0.150 **

Note: ** *p* < 0.01; * *p* < 0.05.

**Table 3:** Linear regression of DASS-21 in during COVID-19 phase group.

Model	Predictors	B(SE)	β	t	*p*
	Constant	53.20 (6.25)	-	8.52	0.001
DASS-21	Gender (Female = 0, Male = 1)	−2.92 (1.10)	−0.130	−2.66	0.01
	Age	−0.293 (0.299)	−0.047	−0.979	0.328
	Social connectedness	−0.482 (0.052)	−0.432	−9.24	0.001
	Social assurance	−0.357 (0.070)	−0.237	−5.12	0.001
	COVID-19 (Having = 0, Not = 1)	2.16 (1.09)	0.100	1.99	0.05
R^2^ = 0.533; adjusted R^2^ = 0.284; F (5,346) = 27.02, *p* < 0.001					

**Table 4:** Linear regression of DASS-21 in post-COVID-19 phase group.

Model	Predictors	B(SE)	β	t	*p*
	Constant	45.48 (7.00)	-	6.49	0.001
DASS-21	Gender (Female = 0, Male = 1)	−5.03 (1.29)	−0.193	−3.91	0.001
	Age	0.156 (0.323)	0.024	0.483	0.629
	Social connectedness	−0.613 (0.068)	−0.450	−9.02	0.001
	Social assurance	−0.100 (0.081)	−0.062	−1.23	0.220
	COVID-19 (Having = 0, Not = 1)	−0.878 (1.23)	−0.035	−0.712	0.477
R^2^ = 0.526; adjusted R^2^ = 0.277; F (5,312) = 23.50, *p* < 0.001					

## Data Availability

The data presented in this study are available on request from the corresponding author. The data are not publicly available due to privacy considerations.
